# Mortality following unemployment in Canada, 1991–2001

**DOI:** 10.1186/1471-2458-13-441

**Published:** 2013-05-04

**Authors:** Cameron A Mustard, Amber Bielecky, Jacob Etches, Russell Wilkins, Michael Tjepkema, Benjamin C Amick, Peter M Smith, Kristan J Aronson

**Affiliations:** 1 Institute for Work & Health, 481 University Ave, Suite 800, Toronto, Ontario, Canada; 2 Dalla Lana School of Public Health, University of Toronto, Toronto, Ontario, Canada; 3 Department of Epidemiology and Community Medicine, University of Ottawa, Ottawa, Ontario, Canada; 4 Health Analysis Division, Statistics Canada, Ottawa, Ontario, Canada; 5 School of Public Health, University of Texas, Houston, Texas, USA; 6 School of Public Health and Preventive Medicine, Monash University, Melbourne, Australia; 7 Department of Community Health and Epidemiology, Queen’s University, Kingston, Ontario, Canada

**Keywords:** Unemployment, Mortality, Occupational health

## Abstract

**Background:**

This study describes the association between unemployment and cause-specific mortality for a cohort of working-age Canadians.

**Methods:**

We conducted a cohort study over an 11-year period among a broadly representative 15% sample of the non-institutionalized population of Canada aged 30–69 at cohort inception in 1991 (888,000 men and 711,600 women who were occupationally active). We used cox proportional hazard models, for six cause of death categories, two consecutive multi-year periods and four age groups, to estimate mortality hazard ratios comparing unemployed to employed men and women.

**Results:**

For persons unemployed at cohort inception, the age-adjusted hazard ratio for all-cause mortality was 1.37 for men (95% confidence interval (CI): 1.32-1.41) and 1.27 for women (95% CI: 1.20-1.35). The age-adjusted hazard ratio for unemployed men and women was elevated for all six causes of death: malignant neoplasms, circulatory diseases, respiratory diseases, alcohol-related diseases, accidents and violence, and all other causes. For unemployed men and women, hazard ratios for all-cause mortality were equivalently elevated in 1991–1996 and 1997–2001. For both men and women, the mortality hazard ratio associated with unemployment attenuated with age.

**Conclusions:**

Consistent with results reported from other long-duration cohort studies, unemployed men and women in this cohort had an elevated risk of mortality for accidents and violence, as well as for chronic diseases. The persistence of elevated mortality risks over two consecutive multi-year periods suggests that exposure to unemployment in 1991 may have marked persons at risk of cumulative socioeconomic hardship.

## Background

Socioeconomic gradients in health, disability and mortality continue to exist in developed economies, including Canada [1]. Experiences and exposures arising from labour force participation are an important potential contributor to the pervasive socioeconomic gradient in life expectancy, where workers in low-skill and low-wage occupations have higher mortality than workers in high-skilled, high-wage occupations [2-6]. Involuntary loss of employment is one such potential exposure. A substantial body of research estimates the consequences to individual physical and mental health of (as well as behavioral responses to) exposure to unemployment [2,7-13]. Moreover, many developed economies are experiencing their sharpest increases in unemployment in more than 50 years, renewing concerns about the impact of unemployment on the health of working age adults [14-17].

Research on the relationship between unemployment and health examines a range of hypotheses concerning pathways and mechanisms for work-related health effects, ranging from the economic consequences of job loss to the disruptions of social ties, by which involuntary loss of employment might lead to disturbance in physical and mental health and function [13,14,18,19]. The evidence of health effects arising from unemployment is sufficiently robust to conclude that a portion of the association is causal: nonetheless, confirming a causal attribution of health consequences following unemployment to the direct or indirect effects of the exposure to unemployment poses a significant challenge [20]. In settings where working-age adults in poorer health are more likely to become unemployed, an association between unemployment and poor health may be primarily due to health selection into unemployment [21]. Long-duration mortality follow-up studies have been valuable in estimating the magnitude of potential health selection effects, by comparing mortality rates in the early follow-up period to mortality rates in a later follow-up period [7,22]. An alternate research design contrasts the relationship between unemployment and mortality in periods of low and high unemployment, under the hypothesis that those who are unemployed during periods of low unemployment are more likely to have pre-existing ill-health than those who are unemployed during periods of high unemployment [23].

Most developed countries use large, nationally-representative population-based cohort studies of mortality to monitor population health, linking records for national census respondents to vital statistics death registrations [2,24-27]. In this study, we relied on a census-based cohort established by linking a 15% sample of 1991 census respondents to the Canadian Mortality Data Base to describe, for the first time in Canada, the association between unemployment and all-cause and cause-specific mortality for a broadly representative sample of occupationally-active adults in Canada [1]. We had three hypotheses: 1) the relative risk of all-cause mortality among the unemployed would be greater in the initial years following unemployment compared to later years, 2) the relative risk of mortality among the unemployed would be greater for causes of death which are more likely to be acute consequence of exposure (such as accidents, violence and alcohol-related causes) and 3) there would be no difference by age in the relative risk of mortality among the unemployed.

## Methods

### Study design

The study is based on a cohort consisting of a 15% sample of the non-institutionalized population of Canada aged 25 years and over at the time of cohort inception in 1991. The cohort was followed for mortality until the end of 2001.

### Study population and study sample

The Canadian census mortality follow-up study is broadly representative of the non-institutional population of Canada in 1991 [1]. We selected cohort members from among the approximately 20% of households who completed a “long-form” census questionnaire in 1991. To ascertain date and cause of death over the follow-up period (from 4 June 1991 to 31 December 2001), we linked records for long-form census respondents to mortality records. For the specific purpose of this study, reporting on the association between unemployment and the risk of mortality, we restricted the study sample to individuals aged 30 to 69 at baseline who had worked one or more weeks in the period 1 January 1990 through 3 June 1991. This represented 1,488,800 persons who were employed on census day (826,600 men and 662,200 women), and 110,800 who were unemployed (61,400 men and 49,400 women).

### Measures

In this report, we describe the relationship between unemployment and mortality by occupational categories, where occupations were grouped into five ordinally-ranked occupational skill levels (professional, managerial, skilled/technical/supervisory, semi-skilled, unskilled) based on the 1990 National Occupational Classification [28]. We also describe the relationship between unemployment and mortality according to baseline age, marital status, immigration status, educational attainment, and industry.

The underlying cause of death is coded to the World Health Organization’s *International Classification of Diseases, Ninth Revision* (ICD-9) [29] for deaths occurring in the period 1991 through 1999, and to the *Tenth Revision* (ICD-10) [30] for deaths occurring in 2000 or 2001. We classified causes of death as malignant neoplasms (stomach, lung, intestine and rectum, other cancers), circulatory diseases (acute myocardial infarction, other ischaemic heart disease, cerebro-vascular disease, other circulatory diseases), respiratory diseases, alcohol-related diseases, accidents and violence (suicide, homicide, traffic accidents, falls, other accidents and violence), and all other causes. Details of our groupings of causes of death are provided in Appendix.

### Statistical analysis

For each member of the cohort, we calculated person-days of follow-up from the beginning of the study (census day, 4 June 1991) to the date of death, emigration (ascertained from the name file and known for 1991 only), or end of the study (31 December 2001). Person-days of follow-up were then divided by 365.25 to get person-years at risk.

Age-standardized mortality rates were calculated by the direct method, with 1991 mid-year population estimates used as the standard population. We used cox proportional hazards models to estimate mortality hazard ratios comparing the unemployed to the employed. Models were estimated for all-cause and cause-specific mortality. Men and women were modelled separately. Models were also estimated for two consecutive periods of follow-up time (1991–1996, and 1997–2001) and for four age groups (30–39, 40–49, 50–59, 60–69 years at baseline). All models were estimated first with age-adjustment only (age in years), and then adjusted for age, marital status, immigration status, occupational skill level, educational attainment, and industry.

The Canadian census mortality follow-up study was approved by the Statistics Canada Policy Committee and was reviewed and approved by the Health Sciences I Research Ethics Board of the University of Toronto. At the time analyses reported in this paper were conducted, access to Canadian census mortality follow-up study database was restricted to employees or deemed employees of Statistics Canada.

## Results

The rate of unemployment in 1991 in this cohort of occupationally-active adults aged 30–69 was 6.9% for men and 6.9% for women. For both men and women, the percentage of the labour force unemployed on census day was higher among persons with lower levels of occupational skill, lower levels of educational attainment and more recent immigration to Canada (Tables 1 and 2).

**Table 1:** Person-years, deaths, crude mortality rates, age-standardized mortality rates (ASMR), 1991–2001, and percent unemployed in 1991 by variables included in the analysis, men aged 30–69 at cohort inception, Canada

	**Person-years**	**Deaths**	ASMR*	**95% confidence interval**		**Percent unemployed in 1991**
**Total, men**	9,195,040	41,680	643.6	636.8	−649.8	6.9
**Employment status**						
Employed	8,562,680	37,974	632.3	624.5	- 640.3	0.0
Unemployed	632,360	3,706	796.2	762.3	- 831.6	100.0
**Occupational skill level**						
Professional	1,245,710	3,851	491.9	473.1	- 511.4	2.6
Managerial	1,436,590	5,377	549.5	530.9	- 568.7	2.9
Skilled/Technical/Supervisory	2,400,490	12,014	719.4	702.5	- 736.6	9.0
Semi-skilled	3,240,000	14,675	637.4	625.0	- 650.1	7.2
Unskilled	872,240	5,763	795.3	771.7	- 819.7	12.7
**Educational attainment**						
Less than secondary graduation	2,665,220	18,578	751.9	739.5	- 764.5	10.1
Secondary graduation	3,651,220	15,012	645.0	631.1	- 659.3	7.1
Postsecondary diploma	1,265,390	3,808	541.3	518.1	- 565.4	4.9
University degree	1,613,200	4,282	446.7	430.4	- 463.6	2.8
**Marital status**						
Legally married	7,176,880	31,000	587.4	579.4	- 595.5	5.8
Common-law	625,830	2,279	846.6	785.3	- 912.7	10.3
Widowed	52,010	830	954.0	868.5	- 1,048.0	8.9
Separated	183,020	1,088	861.3	796.2	- 931.7	10.2
Divorced	330,430	2,432	978.3	926.2	- 1,033.4	10.8
Never married	826,860	4,051	951.0	913.7	- 989.8	12.0
**Year of immigration**						
Non-immigrant	7,196,000	33,235	693.7	684.4	- 703.2	6.9
Before 1971	1,044,090	6,177	537.8	522.0	- 554.1	5.8
1971-1980	517,200	1,448	457.6	421.9	- 496.3	6.3
1981-1991	394,430	753	405.0	370.3	- 442.8	10.9
Non-permanent resident	43,320	67	457.9	312.5	- 670.9	10.2
**Industry sector**						
Primary	779,450	4,397	648.5	629.0	- 668.7	8.7
Manufacturing	1,837,110	7,834	637.4	616.3	- 659.2	7.7
Construction	925,060	4,185	657.7	629.6	- 686.9	16.0
Transportation, storage, communication, utilities	1,042,830	4,563	664.6	636.2	- 694.4	5.2
Trade	1,310,150	5,915	660.2	639.6	- 681.4	5.5
Finance, insurance, real estate	897,990	4,006	611.9	591.5	- 633.1	4.5
Government	886,670	3,807	679.6	649.5	- 711.2	4.2
Other service industries	1,515,760	6,973	610.7	593.7	- 628.1	4.9

* ASMR: Age-standardized mortality rate per 100,000 person-years at risk.

**Table 2:** Person-years, deaths, crude mortality rates, age-standardized mortality rates (ASMR), 1991–2001, and percent unemployed in 1991 by variables included in the analysis, women aged 30–69 at cohort inception, Canada

	**Person-years**	**Deaths**	ASMR*	**95% confidence interval**		**Percent unemployed in 1991**
**Total, women**	7,749,960	16,084	338.9	332.6	−345.3	6.9
**Employment status**						
Employed	6,934,000	14,769	335.5	328.0	- 343.2	0.0
Unemployed	515,960	1,315	384.3	353.8	- 417.5	100.0
**Occupational skill level**						
Professional	1,319,460	2,095	278.4	258.6	- 299.8	3.5
Managerial	551,460	1,120	337.4	309.7	- 367.6	4.5
Skilled/Technical/Supervisory	2,760,800	6,237	346.0	334.1	- 358.3	8.4
Semi-skilled	2,061,580	4,443	336.6	323.9	- 349.8	6.1
Unskilled	756,660	2,189	397.9	375.0	- 422.2	11.5
**Educational attainment**						
Less than secondary graduation	1,877,820	5,974	383.2	370.9	- 395.8	9.6
Secondary graduation	2,831,380	5,678	337.5	324.9	- 350.7	7.1
Postsecondary diploma	1,634,960	2,941	313.2	296.6	- 330.8	5.4
University degree	1,105,800	1,491	252.8	233.1	- 274.2	4.2
**Marital status**						
Legally married	5,264,650	9,988	304.9	295.8	- 314.2	6.7
Common-law	508,880	830	379.2	314.6	- 457.0	9.0
Widowed	203,190	1,340	424.4	394.5	- 456.6	7.3
Separated	235,720	599	411.8	364.2	- 465.7	8.4
Divorced	538,630	1,668	406.8	379.0	- 436.6	7.1
Never married	698,880	1,659	399.0	375.0	- 424.6	6.2
**Year of immigration**						
Non-immigrant	5,895,110	12,998	357.9	349.1	- 366.9	6.8
Before 1971	770,350	2,129	300.8	285.1	- 317.3	6.0
1971-1980	441,380	589	243.8	210.3	- 282.5	7.2
1981-1991	312,450	331	221.3	189.3	- 258.7	11.3
Non-permanent resident	30,680	37	348.3	211.5	- 573.5	10.9
**Industry sector**						
Primary	270,450	726	325.5	301.1	- 351.8	7.7
Manufacturing	757,690	1,590	302.7	279.7	- 327.6	12.5
Construction	133,020	314	372.0	320.1	- 432.4	11.5
Transportation, storage, communication, utilities	351,560	670	384.8	334.0	- 443.3	5.9
Trade	1,120,570	2,621	336.8	320.0	- 354.3	8.0
Finance, insurance, real estate	989,910	1,910	352.5	331.3	- 375.1	6.2
Government	621,640	1,346	377.4	346.4	- 411.2	5.8
Other service industries	3,205,130	6,907	335.1	323.9	- 346.8	5.6

* ASMR: Age-standardized mortality rate per 100,000 person-years at risk.

Compared to persons who were employed on census day, the age-adjusted hazard ratio for all-cause mortality over the eleven-year follow-up period for persons unemployed on census day in 1991 was 1.37 for men (95% confidence interval (CI): 1.32-1.41) (Table 3) and 1.27 for women (95% CI: 1.20-1.35) (Table 4). For both men and women, the age-adjusted hazard ratio for all-cause mortality in the first six years of follow-up (1991–1996) was equivalent to the age-adjusted hazard ratio in the last five years of follow-up (1997–2001).

**Table 3:** Mortality hazard ratios (HR) among the unemployed in 1991, by cause of death, controlling for age and for age and all other variables, men aged 30–69 at cohort inception, Canada, 1991–1996, 1997–2001 and 1991–2001

	**1991-1996**							**1997-2001**							**1991-2001**						
	**Deaths**	**Controlling for age**			**Controlling for all variables (1)**			**Deaths**	**Controlling for age**			**Controlling for all variables (1)**			**Deaths**	**Controlling for age**			**Controlling for all variables (1)**		
		**HR**	**95% CI**		**HR**	**95% CI**			**HR**	**95% CI**		**HR**	**95% CI**			**HR**	**95% CI**		**HR**	**95% CI**	
**All causes of death**	17,923	**1.36**	1.29	−1.43	**1.22**	1.15	−1.28	23,757	**1.37**	1.31	−1.43	**1.22**	1.17	−1.28	41,680	**1.37**	1.32	−1.41	**1.22**	1.18	−1.26
**Malignant neoplasms**	7071	**1.19**	1.09	−1.30	**1.10**	1.01	−1.21	10,079	**1.28**	1.19	−1.37	**1.17**	1.09	−1.26	17,150	**1.24**	1.18	−1.31	**1.14**	1.08	−1.21
Stomach cancer	529	**1.54**	1.16	−2.05	**1.45**	1.08	−1.94	732	1.17	0.89	−1.54	1.06	0.80	−1.40	1,261	**1.33**	1.09	−1.61	**1.22**	1.00	−1.49
Lung cancer	2,248	**1.18**	1.01	−1.38	1.01	0.86	−1.18	3,091	**1.44**	1.28	−1.63	**1.19**	1.05	−1.35	5,339	**1.33**	1.21	−1.46	**1.11**	1.01	−1.23
Cancer of intestine and rectum	908	0.92	0.70	−1.21	0.91	0.69	−1.20	1,302	**1.22**	1.00	−1.50	1.18	0.96	−1.45	2,210	1.10	0.93	−1.29	1.07	0.91	−1.26
Other cancers	3,386	**1.21**	1.07	−1.37	**1.18**	1.04	−1.33	4,954	**1.21**	1.09	−1.34	**1.17**	1.05	−1.30	8,340	**1.21**	1.12	−1.31	**1.17**	1.08	−1.27
**Circulatory diseases**	5,474	**1.19**	1.08	−1.32	1.09	0.98	−1.20	7,208	**1.24**	1.14	−1.35	**1.13**	1.03	−1.23	12,682	**1.22**	1.14	−1.30	**1.11**	1.04	−1.18
Acute myocardial infarction	2,405	1.11	0.95	−1.29	1.02	0.87	−1.19	2,716	**1.21**	1.05	−1.40	1.11	0.96	−1.28	5,121	**1.16**	1.05	−1.29	1.07	0.96	−1.18
Other ischaemic heart disease	1,538	1.13	0.93	−1.37	1.03	0.85	−1.25	2,154	**1.31**	1.12	−1.52	**1.17**	1.00	−1.36	3,692	**1.23**	1.09	−1.39	1.11	0.98	−1.25
Cerebro-vascular disease	553	**1.35**	1.00	−1.81	1.17	0.86	−1.58	863	**1.29**	1.01	−1.64	1.19	0.93	−1.52	1,416	**1.31**	1.09	−1.58	1.18	0.98	−1.43
Other circulatory diseases	978	**1.42**	1.14	−1.76	**1.31**	1.05	−1.63	1,475	1.17	0.97	−1.42	1.06	0.88	−1.29	2,453	**1.27**	1.10	−1.46	**1.16**	1.00	−1.34
**Respiratory diseases**	406	**1.50**	1.07	−2.08	1.34	0.95	−1.87	974	**1.44**	1.16	−1.78	1.22	0.98	−1.52	1,380	**1.45**	1.21	−1.74	**1.25**	1.04	−1.51
**Alcohol-related diseases**	400	**2.14**	1.60	−2.85	**1.54**	1.15	−2.07	526	**2.33**	1.82	−2.98	**1.84**	1.43	−2.37	926	**2.25**	1.86	−2.71	**1.70**	1.40	−2.06
**Accidents and violence**	2,382	**2.01**	1.78	−2.26	**1.59**	1.40	−1.80	2,114	**1.88**	1.65	−2.14	**1.51**	1.32	−1.72	4,496	**1.94**	1.78	−2.12	**1.55**	1.42	−1.70
Suicide	943	**1.94**	1.60	−2.35	**1.58**	1.30	−1.93	879	**1.81**	1.47	−2.22	**1.44**	1.17	−1.78	1822	**1.87**	1.63	−2.16	**1.51**	1.31	−1.75
Homicide	87	**2.78**	1.59	−4.85	**2.10**	1.18	−3.73	50	2.17	0.98	−4.83	1.44	0.63	−3.29	137	**2.55**	1.62	−4.03	**1.83**	1.15	−1.94
Traffic accidents	611	**1.67**	1.29	−2.14	**1.35**	1.04	−1.75	454	**1.51**	1.11	−2.05	1.26	0.92	−1.73	1065	**1.60**	1.32	−1.94	**1.31**	1.07	−1.60
Falls	104	**3.68**	2.29	−5.89	**2.80**	1.71	−4.58	163	**1.73**	1.06	−2.83	1.49	0.90	−2.47	267	**2.44**	1.75	−3.42	**2.00**	1.41	−2.83
Other accidents and violence	637	**2.10**	1.67	−2.63	**1.58**	1.25	−2.00	568	**2.31**	1.82	−2.91	**1.81**	1.42	−2.30	1205	**2.20**	1.86	−2.58	**1.69**	1.42	−1.99
**All other causes**	2,590	**1.60**	1.41	−1.82	**1.33**	1.17	−1.51	3,382	**1.58**	1.41	−1.77	**1.36**	1.22	−1.53	5,972	**1.59**	1.46	−1.73	**1.35**	1.24	−1.47

(1) Adjusted for age, occupational skill level, educational attainment, marital status, year of immigration, and industry sector.

See Appendix for grouping of causes of death by ICD codes.

**Table 4:** Mortality hazard ratios (HR) among the unemployed in 1991, by cause of death, controlling for age and for age and all other variables, women aged 30–69 at cohort inception, Canada, 1991–1996, 1997–2001, and 1991-2001

	**1991-1996**							**1997-2001**							**1991-2001**						
	**Deaths**	**Controlling for age**			**Controlling for all variables (1)**			**Deaths**	**Controlling for age**			**Controlling for all variables (1)**			**Deaths**	**Controlling for age**			**Controlling for all variables (1)**		
		**HR**	**95% CI**		**HR**	**95% CI**			**HR**	**95% CI**		**HR**	**95% CI**			**HR**	**95% CI**		**HR**	**95% CI**	
**All causes of death**	6,742	**1.27**	1.16	−1.39	**1.25**	1.14	−1.36	9,342	**1.27**	1.18	−1.37	**1.23**	1.14	−1.32	16,084	**1.27**	1.20	−1.35	**1.24**	1.17	−1.31
**Malignant neoplasms**	4,168	1.11	0.99	−1.25	1.11	0.99	−1.25	5,576	1.10	0.99	−1.22	1.08	0.97	−1.19	9,744	**1.10**	1.02	−1.19	**1.09**	1.01	−1.18
Stomach cancer	131	1.18	0.62	−2.24	1.14	0.59	−2.19	172	0.70	0.34	−1.42	0.67	0.33	−1.37	303	0.90	0.56	−1.45	0.87	0.54	−1.40
Lung cancer	884	1.24	0.97	−1.59	1.14	0.89	−1.46	1,321	**1.45**	1.20	−1.75	**1.33**	1.10	−1.60	2,205	**1.37**	1.18	−1.59	**1.25**	1.08	−1.45
Cancer of intestine and rectum	413	1.20	0.83	−1.72	1.22	0.85	−1.76	551	1.00	0.72	−1.41	0.99	0.71	−1.39	964	1.09	0.85	−1.39	1.09	0.85	−1.40
Other cancers	2,740	1.05	0.91	−1.22	1.08	0.93	−1.26	3,532	1.01	0.88	−1.15	1.01	0.89	−1.16	6,272	1.03	0.93	−1.13	1.04	0.94	−1.15
**Circulatory diseases**	1,152	**1.26**	1.01	−1.55	1.22	0.98	−1.51	1,732	**1.48**	1.26	−1.74	**1.39**	1.18	−1.64	2,884	**1.39**	1.22	−1.58	**1.32**	1.16	−1.50
Acute myocardial infarction	348	1.39	0.95	−2.02	1.36	0.93	−1.98	467	**1.41**	1.02	−1.95	1.31	0.95	−1.81	815	**1.40**	1.10	−1.79	**1.33**	1.04	−1.70
Other ischaemic heart disease	216	1.49	0.94	−2.36	1.39	0.88	−2.22	395	**1.51**	1.08	−2.13	**1.43**	1.02	−2.02	611	**1.50**	1.15	−1.98	**1.42**	1.08	−1.87
Cerebro-vascular disease	323	0.94	0.60	−1.48	0.89	0.56	−1.40	409	**1.59**	1.15	−2.20	**1.54**	1.11	−2.13	732	**1.30**	1.00	−1.69	1.24	0.95	−1.62
Other circulatory diseases	265	1.30	0.84	−2.01	1.32	0.85	−2.04	461	**1.41**	1.03	−1.95	1.32	0.95	−1.82	726	**1.37**	1.06	−1.78	**1.32**	1.01	−1.71
**Respiratory diseases**	163	1.34	0.77	−2.32	1.29	0.74	−2.24	335	**1.74**	1.23	−2.46	**1.65**	1.17	−2.35	498	**1.61**	1.20	−2.15	**1.53**	1.14	−2.06
**Alcohol-related diseases**	95	**2.07**	1.13	−3.79	1.82	0.98	−3.35	112	**2.52**	1.50	−4.23	**2.17**	1.29	−3.67	207	**2.31**	1.56	−3.42	**2.01**	1.35	−2.99
**Accidents and violence**	560	**2.00**	1.56	−2.56	**1.88**	1.46	−2.42	523	**1.44**	1.08	−1.93	**1.42**	1.06	−1.91	1,083	**1.73**	1.43	−2.08	**1.66**	1.37	−2.01
Suicide	192	**2.20**	1.46	−3.30	**2.10**	1.39	−3.17	186	1.42	0.88	−2.32	1.36	0.83	−2.23	378	**1.81**	1.32	−2.47	**1.73**	1.26	−2.37
Homicide (2)	33	-	-	-	-	-	-	23	0.58	0.08	−4.27	0.54	0.07	−4.10	56	0.24	0.03	−1.71	0.22	0.03	−1.60
Traffic accidents	182	1.31	0.78	−2.19	1.24	0.74	−2.09	164	1.09	0.61	−1.97	1.11	0.61	−2.01	346	1.21	0.82	−1.78	1.18	0.80	−1.74
Falls	22	**6.32**	2.57	−15.5	**5.07**	2.03	−12.7	23	1.35	0.32	−5.77	1.38	0.32	−6.01	45	**3.47**	1.67	−7.21	**3.16**	1.50	−6.65
Other accidents and violence	131	**2.78**	1.76	−4.39	**2.60**	1.63	−4.15	127	**2.16**	1.30	−3.60	**2.15**	1.28	−3.62	258	**2.47**	1.76	−3.47	**2.39**	1.69	−3.37
**All other causes**	699	**1.67**	1.31	−2.12	**1.55**	1.22	−1.98	1,176	**1.61**	1.33	−1.95	**1.50**	1.24	−1.82	1,875	**1.63**	1.41	−1.89	**1.52**	1.31	−1.77

(1) Adjusted for age, occupational skill level, educational attainment, marital status, year of immigration, and industry sector.

(2) Hazard ratios could not be estimated from multivariate models.

See Appendix for grouping of causes of death by ICD codes.

Compared to men who were employed on census day, age-adjusted hazard ratios for unemployed men over the eleven-year follow-up period were elevated for deaths due to malignant neoplasms (1.24, 95% CI: 1.18-1.31), circulatory diseases (1.22, 95% CI: 1.14-1.30), respiratory diseases (1.45, 95% CI: 1.21-1.74), accidents and violence (1.94, 95% CI: 1.78-2.12), and all other causes (1.59, 95% CI: 1.46-1.73) (Table 3). For specific causes of accidental and violent death, eleven-year age-adjusted hazard ratios for unemployed men were significantly elevated for suicide, homicide, motor vehicle accidents, falls and other accidental causes of death. The age-adjusted hazard ratio for alcohol-related diseases was also elevated for unemployed versus employed men (2.25, 95% CI: 1.86-2.71). These risk estimates were moderately attenuated following additional adjustment for occupational skill level, educational attainment, marital status, year of immigration, and industry sector.

Compared to women who were employed on census day, age-adjusted hazard ratios for unemployed women were elevated for deaths due to malignant neoplasms (1.10, 95% CI: 1.02-1.19), circulatory diseases (1.39, 95% CI: 1.22-1.58), respiratory diseases (1.61, 95% CI: 1.20-2.15), accidents and violence (1.73, 95% CI: 1.43-2.08), and all other causes (1.63, 95% CI: 1.41-1.89) (Table 4). For specific accidental and violent causes of death, eleven-year age-adjusted hazard ratios for unemployed women were significantly elevated for suicide, falls and other accidental causes of death. The age-adjusted hazard ratio for alcohol-related diseases among unemployed women was also elevated relative to the employed (2.31, 95% CI: 1.56-3.42). These risk estimates were moderately attenuated following additional adjustment for occupational skill level, educational attainment, marital status, year of immigration, and industry sector.

For both unemployed men and unemployed women, hazard ratios for all-cause mortality were equivalently elevated over the two consecutive multi-year follow-up periods (1991–1996 and 1997–2001) in both age-adjusted and fully-adjusted models.

Table 5 reports hazard ratios for unemployed men and women for all-cause and cause-specific mortality for four age groups. For both men and women, there was a distinct attenuation of the mortality hazard ratio associated with unemployment at older ages. At baseline ages 60–69, the fully adjusted hazard ratios for all-cause mortality among the unemployed were not statistically different from the employed. However, at all ages, unemployed men had elevated mortality hazard ratios due to alcohol-related disorders and accidental and violent causes of death.

**Table 5:** Mortality hazard ratios (HR) among the unemployed in 1991, by age group and cause of death, controlling for age and for age and all other variables, men and women aged 30–69 at cohort inception, 1991-2001

	**Men**							**Women**						
		**Controlling for age**			**Controlling for all variables (1)**				**Controlling for age**			**Controlling for all variables (1)**		
	**Deaths**	**HR**	**95% CI**		**HR**	**95% CI**		**Deaths**	**HR**	**95% CI**		**HR**	**95% CI**	
**Ages 30-39**														
All causes of death	5,021	**1.75**	1.61	−1.90	**1.39**	1.27	−1.51	2,466	**1.39**	1.22	−1.59	**1.30**	1.14	−1.49
Malignant neoplasms	1,156	**1.43**	1.18	−1.72	**1.30**	1.07	−1.59	1,336	1.06	0.87	−1.30	1.02	0.84	−1.25
Circulatory diseases	860	**1.39**	1.11	−1.73	1.11	0.89	−1.40	296	**2.10**	1.52	−2.91	**1.87**	1.35	−2.59
Respiratory diseases	57	2.02	0.96	−4.27	1.57	0.73	−3.38	36	1.13	0.35	−3.67	1.04	0.32	−3.42
Alcohol-related diseases	153	**2.69**	1.78	−4.08	**1.94**	1.26	−2.98	39	**2.76**	1.22	−6.25	2.24	0.97	−5.16
Accidents and violence	1,857	**2.02**	1.77	−2.29	**1.54**	1.34	−1.76	462	**1.70**	1.29	−2.25	**1.61**	1.21	−2.13
All other causes	1,091	**1.93**	1.62	−2.29	**1.40**	1.18	−1.68	336	**1.74**	1.27	−2.42	**1.51**	1.09	−2.09
**Ages 40-49**														
All causes of death	9,195	**1.62**	1.51	−1.73	**1.33**	1.23	−1.43	4,773	**1.44**	1.30	−1.59	**1.38**	1.24	−1.52
Malignant neoplasms	3,500	**1.41**	1.25	−1.59	**1.23**	1.09	−1.40	3,134	**1.16**	1.01	−1.33	1.14	0.99	−1.31
Circulatory diseases	2,647	**1.57**	1.37	−1.79	**1.31**	1.14	−1.50	683	**1.99**	1.57	−2.52	**1.77**	1.40	−2.25
Respiratory diseases	163	**1.96**	1.20	−3.20	1.47	0.89	−2.43	97	**2.60**	1.48	−4.58	**2.24**	1.26	−3.98
Alcohol-related diseases	285	**3.12**	2.28	−4.26	**2.12**	1.53	−2.94	66	**3.77**	2.06	−6.92	**3.35**	1.80	−6.22
Accidents and violence	1,409	**1.96**	1.66	−2.32	**1.51**	1.27	−1.79	360	**1.65**	1.17	−2.34	**1.56**	1.10	−2.23
All other causes	1,476	**1.82**	1.54	−2.15	**1.38**	1.16	−1.64	499	**2.14**	1.64	−2.80	**1.98**	1.51	−2.61
**Ages 50-59**														
All causes of death	15,053	**1.36**	1.29	−1.44	**1.22**	1.15	−1.29	5,555	**1.20**	1.09	−1.32	**1.17**	1.07	−1.30
Malignant neoplasms	7,096	**1.27**	1.17	−1.38	**1.16**	1.07	−1.27	3,510	1.10	0.96	−1.24	1.09	0.96	−1.24
Circulatory diseases	4,731	**1.31**	1.18	−1.45	**1.16**	1.05	−1.29	1,043	1.04	0.82	−1.32	1.00	0.79	−1.27
Respiratory diseases	488	**1.90**	1.45	−2.50	**1.55**	1.17	−2.05	208	**1.64**	1.05	−2.55	**1.62**	1.04	−2.53
Alcohol-related diseases	329	**1.63**	1.14	−2.34	**1.23**	0.85	−1.78	79	1.57	0.76	−3.26	1.35	0.65	−2.83
Accidents and violence	825	**1.88**	1.52	−2.33	**1.56**	1.25	−1.94	177	**2.30**	1.51	−3.52	**2.32**	1.51	−3.56
All other causes	1,913	**1.49**	1.28	−1.74	**1.30**	1.11	−1.52	617	**1.61**	1.24	−2.08	**1.51**	1.17	−1.97
**Ages 60-69**														
All causes of death	12,411	**1.07**	1.00	−1.14	1.03	0.96	−1.10	3,290	1.10	0.95	−1.26	1.09	0.95	−1.25
Malignant neoplasms	5,398	1.08	0.98	−1.19	1.03	0.93	−1.14	1,764	1.09	0.90	−1.31	1.08	0.90	−1.31
Circulatory diseases	4,444	0.93	0.83	−1.05	0.91	0.81	−1.03	862	1.16	0.88	−1.51	1.16	0.89	−1.52
Respiratory diseases	672	1.00	0.74	−1.36	0.95	0.70	−1.29	157	1.16	0.63	−2.14	1.12	0.60	−2.08
Alcohol-related diseases	159	**1.82**	1.14	−2.91	**1.66**	1.03	−2.69	23	0.70	0.09	−5.23	0.66	0.09	−4.95
Accidents and violence	405	**1.69**	1.24	−2.30	**1.69**	1.23	−2.32	84	1.00	0.40	−2.47	0.93	0.37	−2.31
All other causes	1,492	**1.28**	1.07	−1.54	**1.21**	1.01	−1.46	423	1.02	0.69	−1.52	1.00	0.67	−1.49

(1) Adjusted for age, occupational skill level, educational attainment, marital status, year of immigration, and industry sector.

Person-years (000) for men: ages 30–39; 3,768.7, 40–49; 2,984.0, 50–59; 1,784.9, 60–69; 657.4. Person-years for women: ages 30–39; 3,279.1, 40–49; 2,545.6, 50–59; 1,266.1, 60–69; 359.1.

See Appendix for grouping of causes of death by ICD codes.

## Discussion

This study describes the incidence of mortality over an eleven-year follow-up period for a cohort established in 1991—a time of historically high unemployment in Canada. During the 1990–1992 recession, the unemployment rate increased from 7.1% in 1989 to 9.8% in 1991 and remained above 9% through 1994 [31].

For both men and women in this cohort, the age-adjusted relative risk of all-cause mortality in the first six years of follow-up was equivalent to that in the last five years of follow-up. The study's first hypothesis, that the relative risk of all-cause mortality among the unemployed would be greater in the initial years following unemployment compared to later years was not supported.

The study's second hypothesis was that the relative risk of mortality among the unemployed would be greater for those causes of death more likely to arise as a result of an acute consequence of exposure to unemployment. This hypothesis was partially supported. For both men and women, the relative risks of death due to accidents, violence and alcohol-related diseases were greater than the relative risks of death due to malignant neoplasms, circulatory or respiratory diseases. However, there was no attenuation of relative risks comparing the first and second multi-year follow-up periods for causes of death that might be more likely to arise as an acute consequence of exposure to unemployment.

Finally, the study's third hypothesis, that there would be no difference by age in the relative risk of mortality among the unemployed was clearly rejected, a finding consistent with a recent meta-analysis of unemployment and all-cause mortality [13].

The mortality risks reported in this study are generally consistent with a recently published long-duration follow-up study of 50,000 Swedish men born in 1949–1951 that had access to selected measures of health behaviors and psychological health status obtained at time of compulsory military conscription testing at age 20 [21]. Employment status was ascertained over the period 1990–1994, when subjects were 40 years of age, with a nine-year follow-up for mortality (1995–2003). The crude hazard ratio for all-cause mortality among those unemployed Swedish men was 1.91 (adjusted: 1.57, 95% CI: 1.29-1.90), compared to 1.62 (adjusted: 1.33, 95% CI: 1.23-1.43) for our study’s unemployed Canadian males aged 40–49 (Table 5). As in the Canadian cohort, in the Swedish cohort there was a persistence of a higher relative risk of all-cause mortality among the unemployed in both the initial four years of follow-up (HR: 2.39, CI: 1.76-3.26) and in the subsequent five years of follow-up (HR: 1.69, CI: 1.33-2.15).

Institutional features of national economies may mediate the magnitude of potential health consequences arising from unemployment. A recent long-duration follow-up study comparing the risk of mortality following unemployment in Germany and the United States over the period 1984 to 2005 reported an association of unemployment and subsequence risk of death in the United States (adjusted relative risk 2.35, 95% CI 1.67-3.34) but not in Germany (adjusted relative risk 1.42, 95% CI: 0.98-2.04) [32]. The authors suggest that the higher risk of mortality for the unemployed in the United States compared with those in Germany may be related to differences between the two countries in employment protections, such as unemployment insurance, and access to health services. The relatively moderate mortality risk associated with unemployment in Canada is consistent with the hypothesis that more comprehensive social protection policies can influence population health outcomes.

### Strengths and limitations

There are a number of important strengths of the Canadian census mortality follow-up study, including a very large sample broadly representative of the non-institutional adult population of Canada in 1991 and the exclusion of persons not in the labour force [13]. There are also a number of important limitations to acknowledge that are characteristic of census follow-up studies. The study did not have access to information on the duration of the unemployment episode nor information on the number of episodes of unemployment in the follow-up period. Both duration and frequency are relevant for understanding the relationship between exposure burden and health consequences and would be important to address in future research. The census questionnaire did not collect information on behavioral risk factors that would be relevant for understanding the degree to which health status may be a risk factor for subsequent unemployment and separately, a risk factor for mortality [13,20,32-34]. We note, however, that long-duration follow-up studies that do adjust for baseline health status only partially account for the elevated risk of mortality following unemployment. For example, an important recent meta-analysis of 42 studies estimating the association between unemployment and all-cause mortality assessed the magnitude of hazard ratios in studies that controlled for health status and studies that did not have information allowing control for health status [13]. The authors concluded that while baseline health status accounts for a portion of the association between unemployment and mortality, pre-existing health status is not the common cause of both unemployment and mortality. In interpreting the measures of mortality risk reported in our study, readers should note that the hazard ratios may be inflated relative to estimates fully adjusted for baseline health status. As a final limitation, we note that Canada’s adoption in 2000 of ICD-10 for the classification of causes of death may have influenced the comparability of results over time for certain causes [35]. However, we believe the potential influence of the change in disease classification standards to be small.

## Conclusions

This study measured individual employment status and followed individuals forward for 11 years to ascertain deaths. Consistent with results reported from other long-duration cohort studies with information on cause-specific mortality, we observed elevated rates for causes of death that may be sensitive to acute effects of unemployment (such as suicide or deaths resulting from falls), as well as for conditions generally assumed to have long latency (such as circulatory diseases or cancer). Elevated mortality risks among unemployed persons for causes of death with both short and long latency, and the persistence over time of elevated mortality risks across many causes of death suggests that the exposure to unemployment observed in 1991 may have marked persons at risk of cumulative socioeconomic hardship [36]. The elevated risk of mortality among persons experiencing unemployment in this long-duration follow-up study are of moderate magnitude when compared to the United States [32], suggesting that the influence of unemployment income security programs and universal entitlement to access to health care may buffer the potential adverse consequences of unemployment in Canada.

## Appendix

Grouping of causes of death by codes of the International Classification of Diseases (ICD), 9th and 10th revisions (Table 6).

**Table 6:** Grouping of causes of death by codes of the International Classification of Diseases (ICD), 9th and 10th revisions

**Cause**		
	**ICD 9th revision**	**ICD 10th revision**
**Malignant neoplasms**	140-239	C
Stomach cancer	150-151	C15, C16
Lung cancer	162	C33, C34
Cancer of intestine and rectum	152-154, 1590	C17-21, C26
Other cancers	Other 140-239	Other C
**Circulatory diseases**	390-459	I
Acute myocardial infarction	410	I21
Other ischaemic heart disease	411-414, 4292	I20, I22-25, I516
Cerebro-vascular disease	430-438	I60-69
Other circulatory diseases	Other 390-459	Other I
**Respiratory diseases**	460-519	J
**Alcohol-related diseases**	291, 303, 3050, 4255, 5710–5713, 5770, 5771, 860	F10, I426, K70, K85, K860, X45
**Accidents and violence**	800-999	V, W, X, Y
Suicide	95	X60-84
Homicide	96	X85-99
Traffic accidents	810-825, 9290	V02-04, V090, V092, V12-14, V190-196, V20-79, V803-805, V810-811, V820-821, V83-86, V870-878, V880-888, V890, V892
Falls	880-888, 9293	W00-19
Other accidents and violence	Other 800-999	Other V, W, X, Y
**All other causes**	Not 140–239, 390–459, 460–519, 800-999	Not C, I, J, V, W, X, Y

## Competing interest

The authors declare that they have no competing interest.

## Authors’ contribution

CM, RW and KA designed the study. AB, JE, PS, BA and MT contributed to the implementation of the study and to the analyses reported in this paper. CM and RW supervised the analysis. The original version of the manuscript was authored by CM and all authors contributed revisions to the final version of the manuscript. All authors read and approved the final version.

## Pre-publication history

The pre-publication history for this paper can be accessed here:

http://www.biomedcentral.com/1471-2458/13/441/prepub
